# Antiinflammatory and Anticancer Properties of *Grewia asiatica* Crude Extracts and Fractions: A Bioassay-Guided Approach

**DOI:** 10.1155/2022/2277417

**Published:** 2022-03-28

**Authors:** Muhammad Qamar, Saeed Akhtar, Ross T. Barnard, Piero Sestili, Zyta M. Ziora, Claudia E. Lazarte, Tariq Ismail

**Affiliations:** ^1^Institute of Food Science and Nutrition, Bahauddin Zakariya University, Multan 60800, Pakistan; ^2^School of Chemistry and Molecular Biosciences, The University of Queensland, Brisbane, QLD, Australia; ^3^Department of Biomolecular Sciences, University of Urbino Carlo Bo, Via “I Maggetti” 26 61029 Urbino (PU), Italy; ^4^Institute for Molecular Bioscience, The University of Queensland, Brisbane, QLD, Australia; ^5^Department of Food Technology, Engineering and Nutrition, Lund University, Sweden

## Abstract

The study was an extension of our earlier work on antiinflammatory and anticancer properties of *G. asiatica* fruit. We aimed to develop a bioassay guided multistep purification technique for producing bioactive fractions of *G. asiatica* crude extracts. Dried fruit powder was sequentially fractionated with 100% dichloromethane, 100% methanol (MeOH), and 50% MeOH. Active extracts were subjected to liquid-liquid partitioning followed by subfractionation using RP-HPLC. Antioxidant, antiinflammatory, and anticancer activities of the fruit extracts, and their potent fractions were evaluated *in vitro*, while identification of compounds from the bioactive fractions was performed by ESI-MS/MS analysis. The amount of the identified compounds present was confirmed using external standards adopting a simple, accurate, and rapid analytical HPLC method. The results showed that 100% and 50% MeOH extracts possessed bioactivity; one of which (the 50% MeOH extract) displayed potent activity in all *in vitro* bioassays. MeOH extract (50%) derived fraction C and hydroalcoholic fraction 5 (GAHAF5) were observed to possess higher antioxidant, antiinflammatory, and *in vitro* anticancer activity. IC_50_ of GAHAF5 against MCF-7, HEp-2, and NCI-H522 cancer cells was recorded as 26.2, 51.4, and 63 *μ*g/mL, respectively. ESI-MS/MS and HPLC analysis identified catechin, chlorogenic acid, caffeic acid, and morin as potential bioactive compounds in the GAHAF5 fraction with concentrations of 1230, 491, 957, and 130 *μ*g/g, respectively. The findings indicated that *G. asiatica* bioactive fractions possessed antiinflammatory activity *in vitro* and were cytotoxic against breast cancer, lung cancer, and laryngeal cancer cell lines.

## 1. Introduction

Worldwide, an increasing rate of mortality linked to cardiovascular complications, diabetes, various forms of cancer, and many other physiological ailments is creating a burden on the healthcare systems. A plethora of literature correlate modifiable lifestyle factors or health behaviors, for example, poor dietary choices, tobacco use, excessive intake of alcohol, and physical inactivity, with an increasing incidence of the aforementioned chronic diseases [1, 2]. Such a situation calls for a paradigm shift in approaches to improving the health of individuals and hence populations [3]. Fruits and vegetables contain nutrient and nonnutrient substances bearing medicinal properties and are traditionally widely accepted for their relevance to the prevention of various health ailments. However, uncertainties in links between consumption of fruits and vegetables and the reduction of risk of chronic disease require the systematic establishment of a clear connection between the nutrient or nonnutrient components and their ability to reduce the risk of disease [4].

Cancer is among the most common of life-threatening diseases. The International Agency for Research on Cancer has reported worldwide an estimation of 19.3 million new cancer cases and almost 10.0 million cancer-related deaths in the year 2020 [5]. According to the Global Cancer Statistics, Asia has the highest share of cancer-related deaths (57.3%). Approximately one-half of all new cancer cases and more than one-half of the overall cancer deaths were reported from Asia. With an estimation of 2.26 million new cases, breast cancer was reported as the leading type of cancers diagnosed, while the highest incident of cancer-related mortalities, i.e., 1.79 million, was accounted for lung cancer [5, 6]. Chemotherapy, radiotherapy, and chemical drugs are commonly utilized cancer therapies; however, the cost of treatment is high, and there are often adverse side effects [7, 8]. For these reasons, plant-based new medicines are being increasingly investigated as possible alternatives to synthetic drugs subject to their efficacy and consumers' safety [9].

More than 60% of anticancer drugs including vinblastine, vincristine, camptothecin, taxol, podophyllotoxin, and combretastatin are reported to be retrieved from natural sources [10]. The importance of plants and fungi is illustrated by the discovery of penicillin, morphine, and aspirin, the semisynthetic acetylated derivatives of natural salicylic acid. Penicillin is an antibiotic, morphine is a pain reliever, and aspirin is a cyclooxygenase inhibitor used as an antipain, antifever, and antiinflammatory drug, as well as in the prevention of strokes and heart attack owing to its antiplatelet potential [11, 12]. However, many plants used in conventional medicinal or healthcare systems still require scientific exploration and validation.

*G. asiatica* L. (*Tiliaceae*), a berry indigenously known as *Phalsa*, is endemic to South Asian countries, mainly Pakistan and India [1]. Ethnic or traditional use of *G. asiatica* fruit is predominantly used as a refreshing, thirst quenching drink in summer. The fruit drink, also known in some countries as “phalsay ka sharbat,” is thought to be tonic, while the fruit and bark infusions of *G. asiatica* are considered demulcent, febrifuge, and diarrheal remedies [12]. The traditional medicinal claims of *G. asiatica* and its secondary metabolites like flavonoids, phenolic acids, and anthocyanins are supported by the documented pharmacological activities, including antiinflammatory, antidiabetic, anticancer, skin health promotive, and reduction of risk of coronary heart disease [13–16].

In an extension of our earlier work “anticancer and anti-inflammatory perspectives of Pakistan's indigenous berry *Grewia asiatica* Linn (*Phalsa*)” [16], there was a need to identify bioactive fractions of *G. asiatica* as a prelude to their potential exploitation in the food and pharmaceutical industry. This study was therefore intended to develop a bioassay-guided sequential extraction method for recovering bioactive rich fractions of *G. asiatica* fruit and to isolate fractions that may possess higher free radical scavenging, antiinflammatory, and *in vitro* cytotoxic activities against human cancer cells.

## 2. Materials and Methods

### 2.1. Plant Material

*Grewia asiatica* fruits known in Pakistan as *Phalsa* were supplied by a local grower (Multan, Punjab, Pakistan), and taxonomic identification was performed by a botanical expert from the Department of Botany, Bahauddin Zakariya University, Multan. The berries were washed to remove dirt, and then the seed-free pulp was extracted with a fine fruit pulper. The fruit pulp was spread onto stainless steel trays with thickness lesser than 4 mm and dehydrated in a commercial dehydrator (Pamico Tech. Pak) at 40°C. The dehydrated fruit pulp was ground to powder in a kitchen scale grinder and stored in an airtight jar at 4°C for further processing.

### 2.2. Solvents and Reagents

HPLC grade solvents (water, methanol), antiinflammatory and anticancer standard drugs (diclofenac sodium, methotrexate), and analytical and preparative HPLC columns (Zorbax-SB-C-18, Agilent) were purchased from the local supplier of Sigma-Aldrich, USA. Analytical grade solvents, antioxidant assays' reagents, and reference standards were procured from Merck, Pakistan.

### 2.3. Ethical Approval

All studies and testing protocols were in line with the ethical codes mentioned in the Declaration of Helsinki, duly approved by the Bioethical Committee, Bahauddin Zakariya University, Multan, Reg. no. 05-18.

### 2.4. Preparation of Extracts

**Figure 1:**
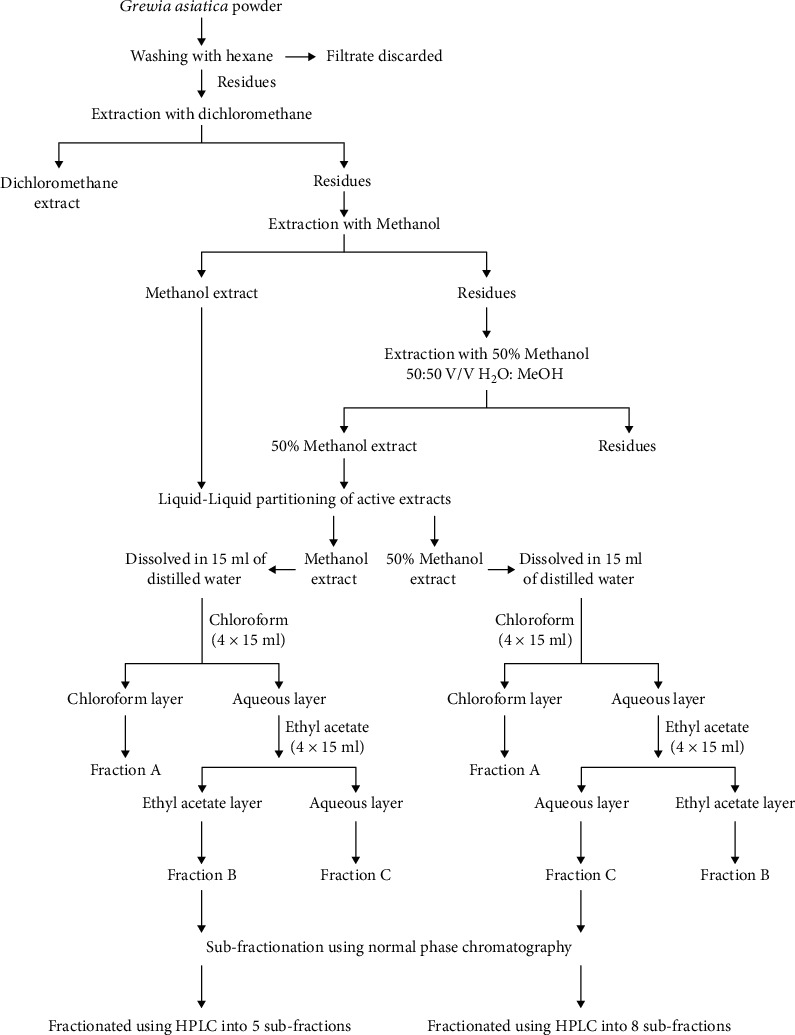
Schematic diagram of *G. asiatica* extract after alcoholic extraction, liquid – liquid partitioning, and subfractionation.

The dried fruit powder was delipidated with hexane and filtered through Whatman No. 1 filter paper. Filtration residues were sequentially fractionated with 100% dichloromethane (DCM), 100% methanol (MeOH), and then 50% MeOH (50 : 50 v/v H_2_O : MeOH) using an orbital shaker. All filtrates were subjected to rotary evaporation (Heidolph, Germany) and stored in an upright ultralow freezer (Sanyo, Japan) at −40°C for bioactive fraction screening assays. The most potent extracts, as evaluated by performance of bioassays, were subjected to liquid-liquid partitioning by solubilization with water (Fraction C) and then partitioned successively, first with chloroform (Fraction A) and then with ethyl acetate (Fraction B), as illustrated in Figure 1.

### 2.5. Determination of Antioxidant Activity

2,2-diphenyl-1-picrylhydrazyl (DPPH) radical scavenging activity of the test samples was conducted in accordance with the method proposed by Alara et al. [17]. The results were calculated as IC_50_ (*μ*g/mL) and compared with the standards, i.e., ascorbic acid and quercetin.

Similarly, hydrogen peroxide (H_2_O_2_) scavenging ability assay was performed with the method followed by Ruch et al. [18] with minor changes. Ascorbic acid and quercetin were used as reference standards, and the results are computed using the following equation:

(1) $$\begin{array}{c} \%\text{inhibition}=\frac{\left(A\text{o}-A\text{s}\right)}{A\text{o}}\times 100 \end{array}$$

where *A*_o_ indicates the absorbance measured for control and *A*_s_ refers to the sample or standard absorbance.

Ferric reducing antioxidant power (FRAP) assay was also performed in accordance with the method followed by Zahin et al. [19]. Ferrous sulfate was used as a reference standard, and the results were computed as mM Fe/g.

### 2.6. In Vitro Antiinflammatory Activity Assays

#### 2.6.1. Membrane Stabilization Assay (Heat-Induced Hemolysis)

Blood was collected in heparinized tubes from healthy human subjects from the cubital vein and centrifuged at 1100 × g for 5 minutes. Blood cells were washed three times with normal saline. The volume of blood was measured and reconstituted as 10% v/v suspension with isotonic buffer solution (10 mM sodium phosphate buffer, pH 7.4) [20, 21].

The membrane stabilization assay was performed in accordance with the method described by Shinde et al. [22]. Briefly, a reaction mixture (2 mL) was prepared by adding 1 mL experimental extracts of different strengths, i.e., 50, 100, 200, and 300 *μ*g/mL to 1 mL (10%) red blood cell (RBC) suspension in isotonic buffer solution (pH 7.4). The tubes containing the reaction mixture were incubated (25 min; 50°C) and cooled. The reaction mixture was centrifuged (1100 × g; 3 min), and the absorbance of the supernatant was checked at 560 nm using a spectrophotometer. Diclofenac sodium was used as a positive control while phosphate buffer as a negative control. The inhibition rate is calculated in accordance with the following equation:

(2) $$\begin{array}{c} \text{Inhibition }\left(\%\right)=\frac{\left(\text{Abs Control}-\text{Abs treated}\right)}{\text{Abs Control}}\times 100. \end{array}$$

#### 2.6.2. Egg Albumin Denaturation Assay

Egg albumin denaturation assay was performed by the method described by Mizushima and Kobayashi [23]. Briefly, 5 mL of the reaction mixture was prepared by mixing egg albumin (0.2 mL), 2.8 mL phosphate buffer (pH 6.5), saline, and 2 mL of extracts of different concentrations, i.e., 50, 100, 200, and 300 *μ*g/mL. The reaction mixture was incubated for a period of 20 min at 40°C followed by heating at 75°C for 5 min in a water bath. The contents of the tubes were cooled, and the absorbance was read spectrophotometrically at 660 nm. Diclofenac sodium and phosphate buffer solution were used as positive and negative control, respectively. Inhibition rates (%) are derived from the following equation:

(3) $$\begin{array}{c} \text{Inhibition }\left(\%\right)=\frac{\left(\text{Abs Control}-\text{Abs treated}\right)}{\text{Abs Control}}\times 100. \end{array}$$

#### 2.6.3. Bovine Serum Albumin Denaturation Assay

Bovine serum albumin denaturation assay was performed in accordance with the method followed by Sakat et al. [21]. Reaction mixture (0.5 mL) was made by adding 0.45 mL of bovine serum albumin and 0.05 mL of experimental extracts of different strengths (50-300 *μ*g/mL). Phosphate buffer (2.5 mL; pH 6.3) was mixed with the reaction mixture, and the contents of the test tubes were incubated for 25 min at 40°C. Cooling was performed, and absorbance was recorded with UV-Vis spectrophotometer (660 nm). Diclofenac sodium and phosphate buffer solution were used as positive and negative controls, respectively. The percent inhibition rates are calculated using the following equation:

(4) $$\begin{array}{c} \text{Inhibition }\left(\%\right)=\frac{\left(\text{Abs Control}-\text{Abs treated}\right)}{\text{Abs Control}}\times 100. \end{array}$$

### 2.7. In Vitro Anticancer Activity

#### 2.7.1. Methyl Thiazolyl Tetrazolium (MTT) Assay

*G. asiatica* extracts and their fractions were evaluated for *in vitro* anticancer potential with the method utilized by Roy et al. [24]. Experimental samples of varying strengths (0.5–200 *μ*g/mL) were prepared in 100 *μ*L dimethylsulphoxide (1% v/v) in microtiter plates. After incubating the microtiter plates (37°C, 48 hours), to each well was added 50 *μ*l of the MTT solution (5 mg/mL). A microplate reader (Tecan, Austria) was used to check the reduction in MTT after a second incubation (37°C, 4 hours) by recording the absorbance at 570 nm. The untreated cells were used as a control against which to measure the effect of experimental extracts on the cell viability. The percent inhibition exhibited on the cell cultures by the test samples is computed using the following equation:

(5) $$\begin{array}{c} \text{Survival }\left(\%\right)=\frac{\left(At-Ab\right)}{\left(Ac-Ab\right)}\times 100, \end{array}$$

where *At*, *Ab*, and *Ac* indicate the sample, blank (complete media without cells), and control absorbance, respectively.

(6) $$\begin{array}{c} \text{Cell inhibition }\left(\%\right)=100-\text{cell survival }\left(\%\right). \end{array}$$

### 2.8. Method Optimization for Fractionation Using RP-HPLC

Fraction C (50% MeOH extract) and Fraction B (100% MeOH extract) were further fractionated using RP-HPLC by dissolving solidified fractions in MeOH (100%) as suggested by Cock [25]. Method optimization for fractionation was performed through Agilent LC technology using an SB-C-18 analytical column (4.6 × 150 mm, 5 *μ*m, Agilent, Germany). The sample was prepared in MeOH at the concentration of 10 mg/mL, and the contents were filtered using 0.45 *μ*m syringe filter. The sample injection limit was set at 5 *μ*L with a flow rate of 0.5 mL/min. Maximum peaks were recorded with acidified (0.1% TFA) water (A) and acidified (0.1% TFA) methanol (B) at 210 (Fraction C) and 280 nm (Fraction B).

### 2.9. RP-HPLC Fractionation (Reverse Phase Chromatography)

RP-HPLC fractionation was performed using Zorbax SB-C18 semipreparative column (25 × 250 mm, 5 *μ*m particle size, Agilent, Germany). The samples were prepared in 100% MeOH as 50 mg/mL; the injection limit was set as 1 mL with a flow rate of 10 mL/min. Eight subfractions were obtained from Fraction C (50% MeOH extract) named *G. asiatica* hydroalcoholic extracts, fractions 1–8, i.e., GAHAF1, GAHAF2, GAHAF3, GAHAF4, GAHAF5, GAHAF6, GAHAF7, and GAHAF8. Accordingly, 5 subfractions were derived from the Fraction B (100% MeOH extracts) termed as *G. asiatica* methanolic fractions 1–5, i.e., GAMF1, GAMF2, GAMF3, GAMF4, and GAMF5.

### 2.10. ESI-MS/MS Analysis

HPLC subfractions with potent activities led to LC-ESI-MS/MS (LTQ XL, Thermo Electron Corporation, USA) analysis for identification of bioactive components adapting guidelines as described earlier by Steinmann and Ganzera [26]. The online software (http://www.chemspider.com/) was used to obtain the structural details of the bioactive compounds identified in the present study.

### 2.11. Quantification Using External Standards

Bioactive compounds tentatively identified earlier were further confirmed by comparing the retention times with external standards and quantified from the percent peak area using HPLC. The chromatograms were obtained at different wavelengths (230, 254, 280, 300, 330 nm). The analytical method for qualitative and quantitative analysis was validated according to the guidelines issued by the proceedings of the International Conference on Harmonization (ICH) for specificity, linearity, accuracy, precision, LOD, and LOQ.

### 2.12. Statistical Analysis

The data were expressed as mean ± SD. One-way analysis of variance (ANOVA) was used to measure statistical differences between the controls and treatments. The data was subsequently analyzed by Dunnett's test. Prism (Graph Pad Software, San Diego, USA) was used to plot the graphs, and *p* values were indicated as follows: ∗*p* < 0.05, ∗∗*p* < 0.01, ∗∗∗*p* < 0.001, and ∗∗∗∗*p* < 0.0001.

## 3. Results and Discussion

### 3.1. Antioxidant Activity

The results presented in Table 1 show that Fraction C and Fraction B possessed the lowest IC_50_ in the DPPH assay, the highest ferric reducing antioxidant power (FRAP), and the maximum radical scavenging in H_2_O_2_ assay compared to their parent extracts, i.e., 50% MeOH and 100% MeOH extract, respectively. Among RP-HPLC subfractions, GAHAF5 of Fraction C (50% MeOH extract) exhibited considerable radical scavenging potential in DPPH (IC_50_ of 29 *μ*g/mL), FRAP (46 mmol/g), and H_2_O_2_ (77%) assays, comparable to the antioxidant activity of standard ascorbic acid or quercetin (Table 1). Likewise, a notable increase in the antioxidant activity was also observed for GAMF3 in comparison to Fraction B (100% MeOH extract). Previously, a methanol extract of *G. asiatica* leaves was reported to hold notable DPPH radical scavenging activity (IC_50_ of 27.3 pg/mL) and nitric oxide radical inhibition activity (IC_50_ of 56.85 *μ*g/mL) when compared with ascorbic acid or quercetin as standards [27]. The strong antioxidant potential of *G. asiatica* was also cited by Mesaik et al. [28], who reported that the 20 ppm flavanol fraction of the fruit produced 85% inhibition of DPPH stable-free radicals. Data on qualitative screening of the *G. asiatica* extracts (Supplementary Table 1) further suggest the fruit as plausible carrier of secondary metabolites such as flavonoids, phenols, and tannins which may serve as active-free radical inhibitors alike other fruits of ethnomedicinal significance including *S. cumini* and *C. carandas.*

### 3.2. In Vitro Antiinflammatory Activity

#### 3.2.1. Heat-Induced Hemolysis (Membrane Stabilization)

The stabilization effect of a drug on human red blood cell membranes (HRBC) against heat and hypotonicity induced lysis is documented as a proxy for antiinflammatory action of a drug [29, 30]. The human red blood cell membrane and lysosomal membrane are similar in composition; therefore, drugs protecting HRBC membrane may also protect against destruction of the lysosomal membrane [31].

Consistent with the above, antiinflammatory activity of *G. asiatica* successive extracts, their partitioned fractions, and RP-HPLC subfractions were studied at varying concentrations (50, 100, 200, and 300 *μ*g/mL). The findings revealed a dose-dependent inhibition of heat-induced hemolysis and improved efficacy of the extracts from crude RP–HPLC subfractions. Among the tested fractions, GAHAF5 anticipated 59% inhibition of heat-induced hemolysis at a dose of 300 *μ*g/mL, whilst the inhibition by 50% MeOH extract and fraction C was 50 and 56%, respectively. Data shown in Table 1 suggest a strong relationship between antioxidant and antiinflammatory activity. A similar study conducted by Moussaid et al. [32] reported a positive correlation between the concentrations of experimental extracts and membrane stabilization. Furthermore, extracts having antioxidant activity considerably inhibited the inflammatory responses. Likewise, 100% MeOH extract, Fraction B, and GAMF3 dispensed at 300 *μ*g/mL exerted 32 and 46% inhibition in heat-induced hemolysis when compared with the normal control, respectively. Earlier, Khanal et al. [33] in their study on antiinflammatory activity of *G. asiatica* fruit extracts—as determined with membrane stabilization assays—reported 81% inhibition at 600 *μ*g/mL, a finding remarkably like ours.

#### 3.2.2. Inhibition of Protein Denaturation (Serum and Egg Albumin)

Denaturation of proteins is a well-documented cause of inflammation, while substantial data is available to confirm the link between denaturation of tissue proteins and the onset of inflammatory complications [34, 35]. Plant extracts, owing to their capacity to react with erythrocyte membrane proteins, may deform the cells [21] and provoke alteration of cell surface charges [36].

Our multistep purification technique produced fractions that positively affected egg albumin denaturation inhibition: indeed, a 56% inhibition of albumin denaturation was observed for crude extracts (50% MeOH extract), and a 63% inhibition was found in the case of the purified successive fraction (GAHAF5). The results further suggested a dose-dependent albumin denaturation inhibitory response that has also been documented for coffee extracts compared with the diclofenac sodium standard [37]. Another study delineated a dose-dependent effect of methanolic extract of *Enicostemma axillare* with a maximum inhibition of 71% observed at 500 *μ*g/mL [38]. Accordingly, moderate inhibition of protein denaturation at 300 *μ*g/mL was observed with pure MeOH extract (36%), Fraction B (44%), and GAMF3 (51%) (Table 1).

A similar inhibitory trend was recorded against serum albumin denaturation assay suggesting that 50% MeOH extract (300 *μ*g/mL) exhibit the highest inhibition of albumin denaturation, i.e., 65%, when compared to other extracts. Among liquid-liquid partitioned fractions, only Fraction C and, from RP-HPLC subtractions, only GAHAF5 outlined inhibitory activity as 70% and 75%, respectively, in albumin denaturation inhibition assay which is slightly more than the parent 50% MeOH extract. A standard drug, diclofenac sodium, offered a potent inhibition of 98%, 96%, and 98% at a dose of 300 *μ*g/mL against heat-induced hemolysis, egg albumin denaturation, and bovine serum albumin denaturation assays, respectively (Table 1).

### 3.3. Anticancer Activity

The crude MeOH extracts (50 and 100%) demonstrated potent to moderate *in vitro* anticancer activity which was further characterized in the successive extracts obtained through bioassay guided fractionation. Bioassay-guided fractionation is an established and effective method for purifying anticancer, antifungal, and antifertility compounds [39–41]. Our results suggest that the 50% MeOH extract derived Fraction C as the fraction exhibiting the highest anticancer activity, with an IC_50_ of 30 *μ*g/mL, 81 *μ*g/mL, 73 *μ*g/mL, 114 *μ*g/mL, and 279 *μ*g/mL against human cell lines derived from breast cancer, lung cancer, laryngeal cancer, epidermal kidney cancer, and cervical cancer, respectively. Since IC_50_ of extracts and fractions against epidermal kidney cancer and cervical cancer were above 100 *μ*g/mL, the values are therefore not tabulated. In the case of breast and laryngeal cancer cell lines, Fraction C demonstrated the lowest IC_50_ when compared with the parent extracts. Similarly, Fraction B originating from 100% MeOH extract exerted considerable cytotoxic activity against breast and lung cancer cell lines. IC_50_ of fraction B against breast cancer and lung cancer were 61 *μ*g/mL and 95 *μ*g/mL, respectively, which are also higher than recorded from the parent extracts (Table 1).

The IC_50_ for 50% MeOH extract against MCF-7 breast cancer cells was up to 35 *μ*g/mL, while lower values were recorded for fraction C and subfraction GAHAF5, i.e., 30 *μ*g/mL and 26 *μ*g/mL, respectively. Parallel trends were observed with lung and laryngeal cancer cell lines, where GAHAF5 exhibited the lowest IC_50_, i.e., 63 *μ*g/mL and 51 *μ*g/mL with lung cancer and laryngeal cancer, respectively. Interestingly, GAMF3 from Fraction B was cytotoxic against the breast cancer cell line (IC_50_ of 52 *μ*g/mL). Previously, M1 fractions retrieved from the parent hexane extract of *Mangifera zeylanica* bark obtained through a series of purification techniques were reported more effective than the parent hexane extract against three cancer cell lines. The referenced study demonstrated a less toxic effect of bioassay guided fraction when compared with the parent extracts on normal mammary epithelial cells [41]. Glaucarubinone, a triterpenoid obtained from the hexane extracts of Brazilian cerrado, was reported biologically active against cancer cell lines of common cancers [42].

### 3.4. ESI-MS/MS Analysis

**Figure 2:**
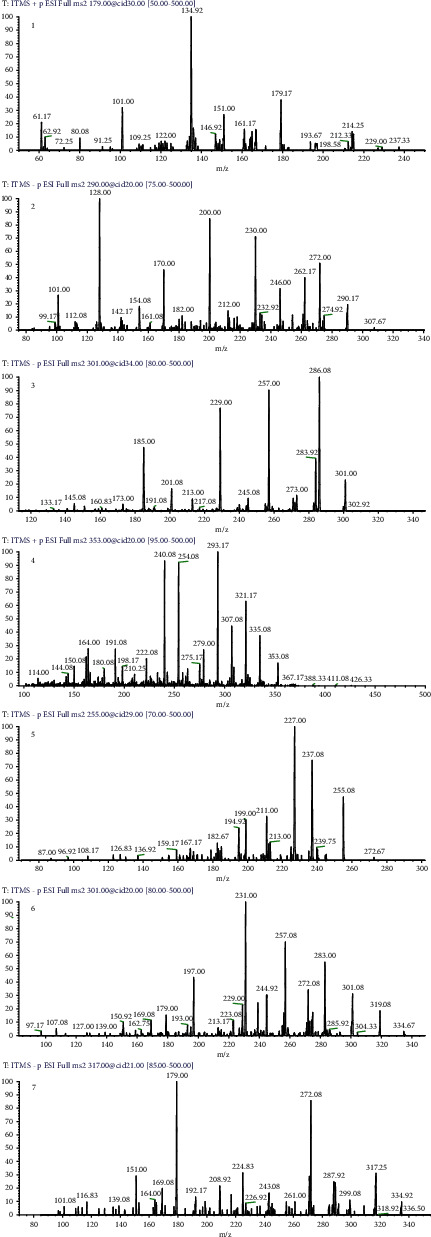
Mass spectras of identified compounds. (1) Caffeic acid; (2) catechin; (3) morin; (4) chlorogenic acid; (5) liquiritigenin; (6) quercetin; (7) myricetin.

The bioactive fraction of *G. asiatica* fruit extracts, i.e., GAHAF5, was identified to contain caffeic acid, morin, catechin, and chlorogenic acid (Figure 2.  Table 2). Mass spectras of compounds that we were unable to identify are also available in Supplementary Figures 1 and 2. Previously, all referred compounds have been cited as antiinflammatory agents [43–46]. Morin appeared to induce cytotoxicity among metastatic breast and lung cancer cells [47, 48]. Caffeic acid was reported to induce apoptosis in breast cancer [49] and lung cancer cells [50]. Chlorogenic acid regulated apoptosis in A549 human lung and breast cancer cells [51]. In addition, catechin was reported to exhibit a significant inhibition in the proliferation of breast [52] and lung cancer [53].

Likewise, liquiritigenin, quercetin, and myricetin were also identified in GAMF3 fraction of our study (Figure 2), while the literature confirms the significant antiinflammatory potential of liquiritigenin [54], myricetin [55], and quercetin [56]. Moreover, liquiritigenin, myricetin, and quercetin were also presented as potent breast cancer inhibitors in some previous findings [57–59].

### 3.5. Quantification of Bioactive Compounds Using External Standards

#### 3.5.1. Specificity Validation

**Figure 3:**
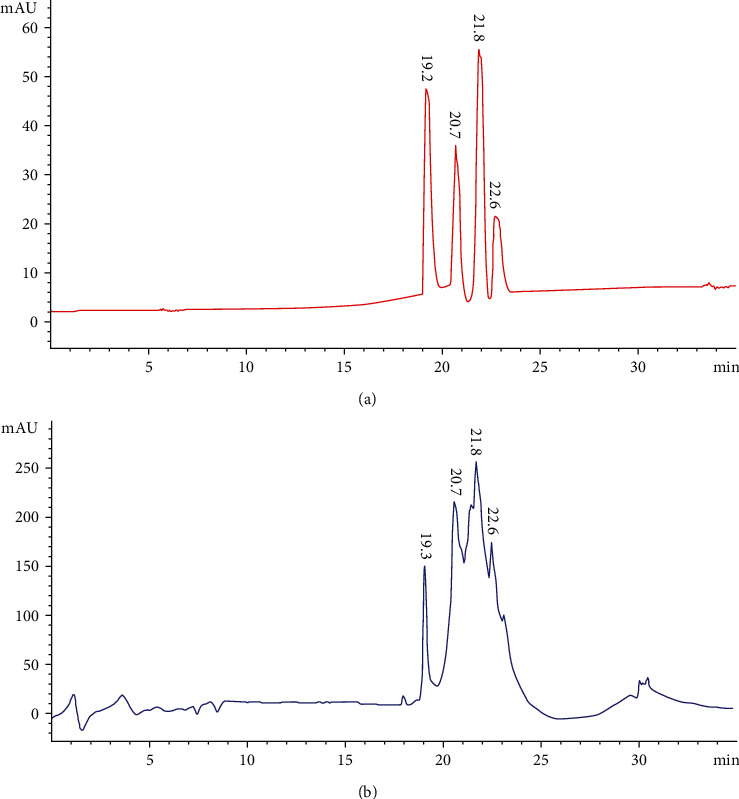
HPLC chromatograms of external standards (A) and GAHAF5 (B) at 300 nm. (1)Catechin; (2) chlorogenic acid; (3) caffeic acid; (4) morin.

**Figure 4:**
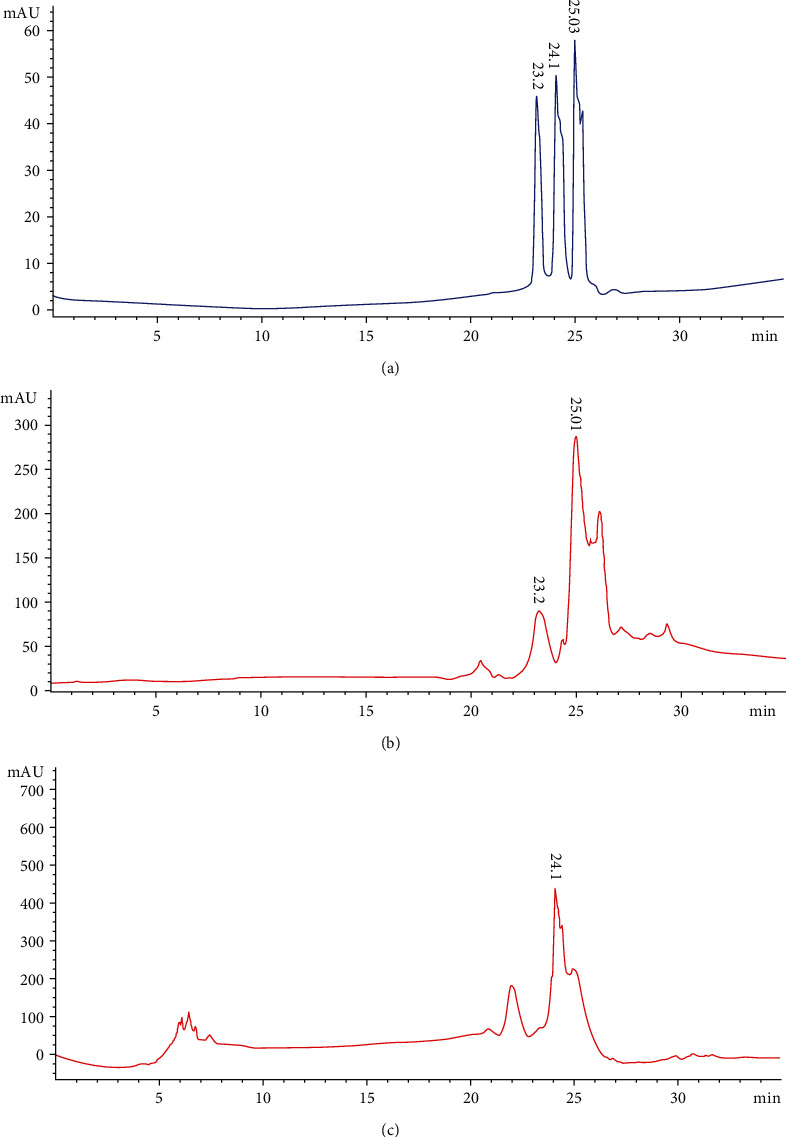
HPLC chromatograms of external standards (a) and GAMF3 at 230 nm (b) and at 230 nm (c): (1) liquiritigenin; (2) quercetin; (3) myricetin.

The analytical method was evaluated for its specificity by comparing the retention times and mass spectra of external standards with HPLC chromatogram peaks of *G. asiatica* fractions. Firstly, a visible separation effect was obtained for catechin, chlorogenic acid, caffeic acid, and morin eluted at 19.3, 20.7, 21.8, and 22.6 minutes, respectively, from standard solution and from GAHAF5 (Figure 3). Likewise, it has been apparent from Figure 4 that well-distinguished peaks of myricetin, quercetin, and liquiritigenin were established and eluted at 24.1 minutes (myricetin), 25.0 minutes (quercetin), and 23.2 minutes (liquiritigenin). Chromatograms of GAMF3 also showed visible peaks of quercetin, liquiritigenin, and myricetin at similar retention times.

#### 3.5.2. Quantification Parameters

The current method was validated for linearity performance using calibration curves. Seven calibration curves in triplicate were established. The calibration curves of catechin, chlorogenic acid, caffeic acid, morin, myricetin, quercetin, and liquiritigenin were linear between 46.8 and 1500, 7.8 and 500, 7.8 and 500, 46.8 and 1500, 12.5 and 200, 1.6 and 25, and 3.9 and 250 *μ*g/mL with a regression coefficient (*r*^2^) between 0.9985 and 0.9995. Regression data, LODs, and LOQs for all seven standard substances are given in Table 3.

#### 3.5.3. Accuracy Validation

**Figure 5:**
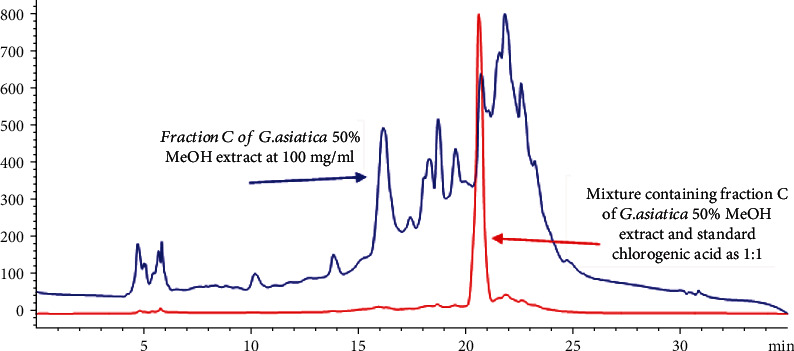
Overlay chromatogram of Fraction C of *G. asiatica* 50% MeOH extract and mixture as 1 : 1.

**Figure 6:**
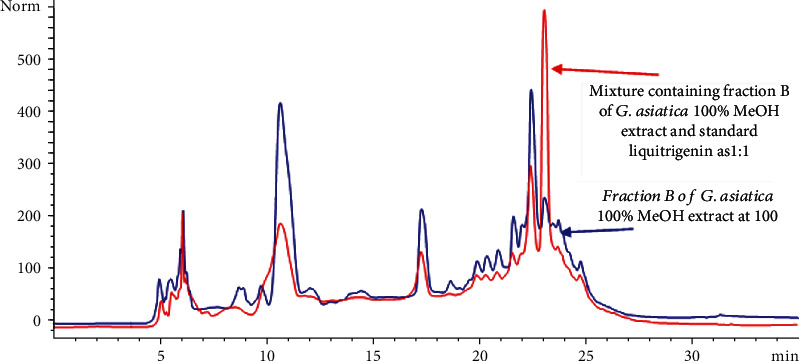
Overlay chromatogram of Fraction B of *G. asiatica* 100% MeOH extract and mixture as 1 : 1.

Bioactive fractions of *G. asiatica*, i.e., GAHAF5 and GAMF3, were spiked with standards of varying concentrations, i.e., 200, 400, and 500 *μ*g/mL. Recovery rates of the compounds listed in Table 4 were within the range of 98.1 ± 1.01-104.1 ± 1.53% with a percent variation coefficient between 1.02 and 3.65% (Table 4). HPLC overlay chromatograms were also developed to identify the percentage recovery of the bioactive compounds of *G. asiatica.* Figures 5 and 6 represent overlay chromatograms of *G. asiatica* 50% MeOH extract, Fraction *C* with standard chlorogenic acid (1 : 1), and 100% MeOH derived Fraction B with standard liquiritigenin (1 : 1), respectively.

#### 3.5.4. Precision Validation

Instrumental precision was determined by replicate analysis of external standards. The results of intraday and interday analysis of all seven standard compounds showed a high precision with coefficient of variation below 2%, which demonstrates the good precision of our analytical investigation (Table 5).

#### 3.5.5. Quantification Analysis

In this study, the chromatogram peak heights of both fractions (GAHAF5, GAMF3) were compared with those of external standards. The peak height with similar retention times indicates the presence of the respective compounds whose levels were quantified using calibration curves obtained with the corresponding external standards. The detected levels of catechin, chlorogenic acid, caffeic acid, and morin in GAHAF5 were 1230, 491, 957, and 130 *μ*g/g, respectively. Likewise, the levels of liquiritigenin, quercetin, and myricetin in GAMF3 were 217, 591, and 24 *μ*g/g. Earlier, quercetin was detected as 2.4 ng/*μ*l and 4.28 ng/*μ*l in *G. asiatica* callus and leaf extracts, respectively [60]. More recently, LC-QToF-MS analysis of *G. asiatica* extracts tentatively identified and provided relative abundance of some bioactive compounds like quercetin, myricetin, umbelliferone, isovitexin, petunidin, kaempferol, and morin as 0.44, 4.87, 0.10, 0.33, 0.60, 0.87, and 4.25 *μ*g/g, respectively [61].

## 4. Conclusion

Bioassay-guided fractionation of *G. asiatica* fruit extracts proved to be promising as a means to obtain bioactive fractions bearing significant *in vitro* antioxidant, antiinflammatory, and *in vitro* anticancer activity. Bioactive compound characterization data suggest that chlorogenic acid, caffeic acid, gallic acid, and morin represent the key components of GAHAF5 responsible for the cytotoxic effect on breast, lung, and laryngeal cancer cells. Bioactive fractions from the *G. asiatica* fruit extracts as recovered in this study are potential drug leads and warrant additional testing and determination of therapeutic index *in vivo*.

## Figures and Tables

**Table 1:** *In vitro* antioxidant and antiinflammatory potential of *G. asiatica.*

Fruit samples	*In vitro* antioxidant assays			*In vitro* antiinflammatory assays (% inhibition at 300 *μ*g/mL)			Anticancer assay (IC_50_*μ*g/mL)		
	DPPH (IC_50_*μ*g/mL)	FRAP (mmol/g)	H_2_0_2_ (%)	Heat-induced hemolysis	Egg albumin denaturation	Bovine serum albumin denaturation	MCF-7	HEp-2	NCI-H522
100% DCM	153 ± 2.3	14 ± 0.2	33 ± 1.44	12 ± 0.2^ns^	17 ± 0.2^ns^	21 ± 0.3^ns^	175	257	232
100% MeOH	77 ± 1.1	27 ± 0.7	43 ± 0.4	32 ± 0.1^∗^	36 ± 0.1^∗^	44 ± 1.1^∗^	86	126	107
Fraction B	62 ± 0.2	39 ± 0.9	51 ± 1.1	39 ± 0.1^∗^	44 ± 0.9^∗^	49 ± 1.1^∗^	61	141	95
GAMF3	56 ± 0.3	34 ± 0.2	49 ± 0.1	46 ± 1.1^∗^	51 ± 0.1^∗^	56 ± 0.3^∗∗^	52	162	67
50% MeOH	41 ± 1.0	43 ± 0.6	73 ± 0.6	50 ± 0.2^∗^	56 ± 0.0^∗∗^	65 ± 0.0^∗∗^	35	80	73
Fraction C	37 ± 1.2	44 ± 0.2	70 ± 0.1	56 ± 0.1^∗∗^	61 ± 0.2^∗∗^	70 ± 0.2^∗∗^	30	73	81
GAHAF5	29 ± 0.1	46 ± 1.1	77 ± 0.2	59 ± 0.2^∗∗^	63 ± 1.2^∗∗^	75 ± 1.1^∗∗∗^	26	51	63
Ascorbic acid	21 ± 0.2	56 ± 1.1	78 ± 0.2	—					
Quercetin	18 ± 0.5	63 ± 0.1	82 ± 0.3	—					
Diclofenac sodium	—			98 ± 0.02^∗∗∗∗^	96 ± 0.02^∗∗∗∗^	98 ± 0.01^∗∗∗∗^	—		
Methotrexate				—					

Values are means ± S.D. DCM extract =100% dichloromethane extract; 100% MeOH extract =100% methanolic extract; GAMF = *G. asiatica* methanolic fraction; 50% MeOH extract = methanol : water (50 : 50 v/v); GAHAF = *G. asiatica* hydroalcoholic fraction; DPPH = 2,2-diphenyl-1-picrylhydrazyl; FRAP = ferric reducing antioxidant power; TPTZ = 2,4,6-tripyridyl-s-triazine; H_2_O_2_ = hydrogen peroxide ∗*p* < 0.05, ∗∗*p* < 0.01, ∗∗∗*p* < 0.001, ∗∗∗∗*p* < 0.0001.

**Table 2:** ESI-MS/MS identification of bioactive compounds from *G. asiatica* different fruit fractions.

Fractions	Average mass	ESI-MS/MSn (ions)	Identification	Chemical formula	References
GAHAF5	179	179, 161	Caffeic acid	C_9_H_8_O_4_	[62]
	290	290, 272.08, 246	Catechin	C_15_H_14_O_6_	[62]
	302	302, 286.07	Morin	C_15_H_10_O_7_	[61]
	354	353.25, 191	Chlorogenic acid	C_16_H_18_O_9_	[62]
GAMF3	256	255, 237	Liquiritigenin	C_15_H_12_O_4_	[63]
	301	301, 272, 179	Quercetin	C_15_H_10_O_7_	[64]
	317	317, 179	Myricetin	C_15_H_10_O_8_	[64]

**Table 3:** Quantification parameters of three phenolic acids, and four flavonoids from *G. asiatica* different fruit fractions.

Fractions	Compounds name	Wavelength	LOD	LOQ	Linear range (*μ*g/mL)	*r* ^2^	R_t_ min	Concentration (*μ*g/g)
GAHAF5 (50% MeOH extract)	Catechin	300 nm	3.10	9.60	46.8-1500	0.9994	19.3	1230
	Chlorogenic acid		0.2	0.5	7.8-500	0.9995	20.7	491
	Caffeic acid		2.7	8.4	7.8-500	0.9991	21.8	957
	Morin		1.3	3.24	46.8-1500	0.9993	22.6	130
GAMF3 (100% MeOH extract)	Myricetin	330 nm	0.9	2.16	12.5-200	0.9985	24.1	217
	Quercetin	230 nm	1.1	2.75	1.6-25	0.9991	25.0	591
	Liquiritigenin		2	4.90	3.9-250	0.9987	23.2	24

LOD = limit of detection; LOQ = limit of quantification; *r*^2^ = regression coefficient. Rt min = retention time in minutes; GAMF3 = *Grewia asiatica* methanolic fraction; GAHAF5 = *Grewia asiatica* hydroalcoholic fraction; 100% MeOH = 100% methanolic extract; 50% methanolic extract (50/50 H_2_O: MeOH).

**Table 4:** Accuracy validation of analytical method three phenolic acids and four flavonoids from *G. asiatica* different fruit fractions.

Marker substance	Standard additions *μ*g/mL	% recovery			Mean	% CV
		Day 1	Day 2	Day 3		
Catechin	200	97.1	97.9	99.4	98.12 ± 1.16	1.18
	400	101.2	98.7	100.7	100.2 ± 1.32	1.31
	500	101.3	102.9	100.2	101.4 ± 1.35	1.33
Chlorogenic acid	200	97.2	97.9	99.1	98.06 ± 1.01	1.02
	400	99.3	99.8	102.4	100.5 ± 1.16	1.15
	500	103.4	101.4	102.9	102.5 ± 1.04	1.01
Caffeic acid	200	101.3	99.1	104.3	101.5 ± 2.61	2.57
	400	103.5	104.3	100.5	102.7 ± 2.00	1.94
	500	100.5	103.3	105.6	103.1 ± 2.55	2.47
Morin	200	97.4	98.0	100.5	98.63 ± 1.64	1.66
	400	99.9	97.3	101.2	99.46 ± 1.98	1.99
	500	100.9	103.4	105.3	103.2 ± 2.20	2.13
Quercetin	200	99.0	103.3	97.8	100.03 ± 2.89	2.88
	400	98.9	101.4	96.2	98.83 ± 2.60	2.63
	500	104.5	105.4	102.4	104.1 ± 1.53	1.46
Myricetin	200	96.0	100.3	101.8	99.36 ± 3.01	3.02
	400	99.9	102.4	97.2	99.83 ± 2.60	2.60
	500	99.5	101.4	104.4	101.7 ± 2.47	2.42
Liquiritigenin	200	98.0	100.3	96.8	98.36 ± 1.77	1.79
	400	97.9	100.4	105.2	101.1 ± 3.70	3.65
	500	100.5	103.4	102.4	102.1 ± 1.47	1.43

Values shared are mean ± SD of triplicates. Percent coefficient of variation (% CV); (SD/Mean) × 100.

**Table 5:** Precision validation of analytical method of three phenolic acids and four flavonoids from *G. asiatica* different fruit fractions.

Marker substance	Theoretical concentration (*μ*g/mL)	Intraday precision (*n* = 3)		Interday precision (*n* = 9)	
		Measured concentration (*μ*g/mL)	CV (%)	Measured concentration (*μ*g/mL)	CV (%
Catechin	400	403.6 ± 0.20	0.04	401.4 ± 0.79	0.19
Chlorogenic acid	230	231 ± 1.75	0.75	230.9 ± 0.24	0.10
Caffeic acid	100	102.4 ± 0.28	0.27	100.9 ± 0.31	0.30
Morin	500	498.4 ± 0.57	0.11	499.2 ± 1.13	0.22
Myricetin	150	151.8 ± 1.15	0.75	150.9 ± 0.83	0.55
Quercetin	20	19.6 ± 0.22	1.1	19.9 ± 0.01	0.05
Liquiritigenin	200	201 ± 0.74	0.36	200.9 ± 0.39	0.19

^a^Values shared are mean ± SD of triplicates ^b^Values shared are mean ± SD of triplicates for 3 days. Percent coefficient of variation (% CV); (SD/Mean) × 100.

## Data Availability

The data supporting the conclusion of this study are included and are available within the article.

## Abbreviations

RP-HPLC: Reverse phase high performance liquid chromatography
ESI-MS/MS: Electrospray ionization mass spectrometry
MCF-7: Breast cancer cell line
HEp-2: Laryngeal cancer cells
NCI-H522: Lung cancer cell line
DPPH: 2,2-diphenyl-1-picrylhydrazyl
FRAP: Ferric reducing antioxidant power
H_2_O_2_: Hydrogen peroxide
MTT: Methyl thiazolyl tetrazolium
GAMF: *Grewia asiatica* methanolic fraction
GAHAF5: *Grewia asiatica* hydroalcoholic fraction
100% MeOH: 100% methanolic extract
50/50 H_2_O: MeOH 50% hydromethanolic extract
TFA: Triflouroacetic acid
LOD: Limit of detection
LOQ: Limit of quantification
*r* ^2^: Regression coefficient
Rt min: Retention time in minutes.

## Supplementary Materials

Supplementary Materials Supplementary Figures 1 and 2: raw data of ES-MS/MS analysis of GAHAF5 and GAMF3 fractions showing spectras of known and unknown compounds. Supplementary Table 1: qualitative screening of *G. asiatica* fruit extracts showing presence of secondary plant metabolites.
