# Supplementary material for: Antiinflammatory and Anticancer Properties of Grewia asiatica Crude Extracts and Fractions: A Bioassay-Guided Approach
Source: Biomed Res Int. 2022 Mar 28;2022:2277417. doi: 10.1155/2022/2277417 (PMC8979695; doi:10.1155/2022/2277417)
Supplement: Supplementary Materials — Supplementary Figures 1 and 2: raw data of ES-MS/MS analysis of GAHAF5 and GAMF3 fractions showing spectras of known and unknown compounds. Supplementary Table 1: qualitative screening of G. asiatica fruit extracts showing presence of secondary plant metabolites. [file 2277417.f1.zip › Supplementary figure 1.pdf]

Supplementary figure 1 (a – h): ESI-MS/MS analysis of GAHAF5

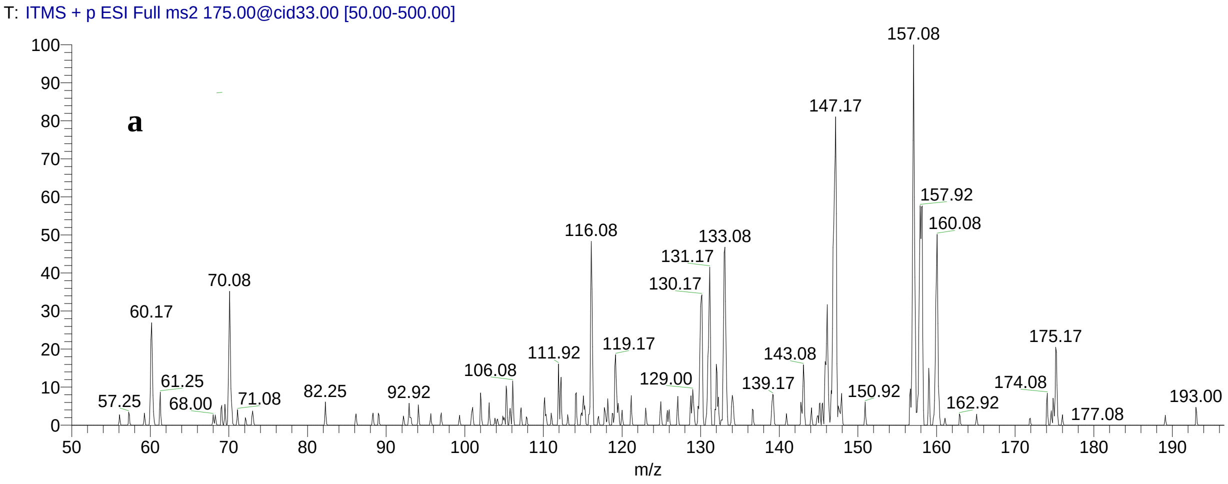

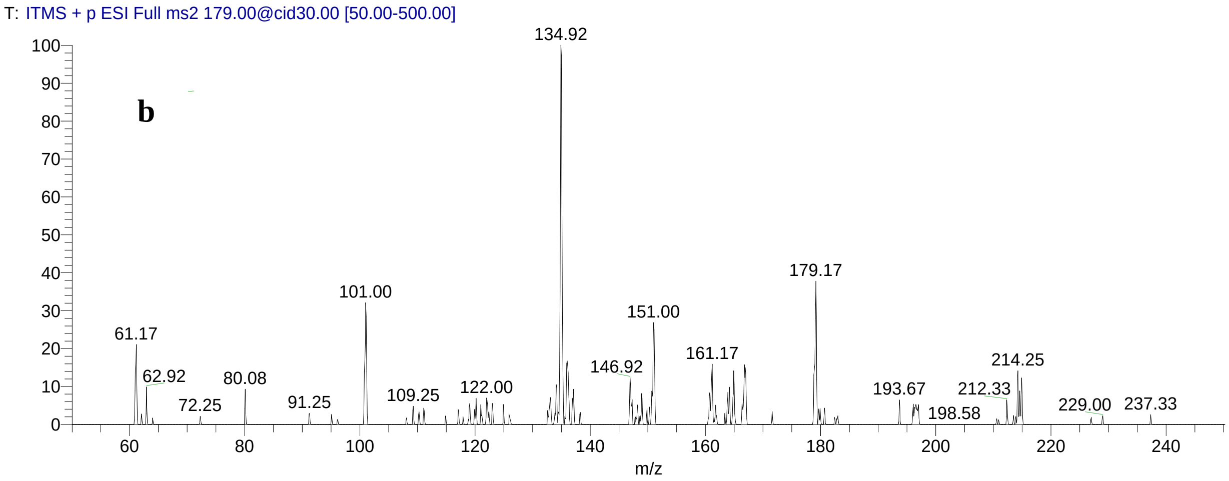

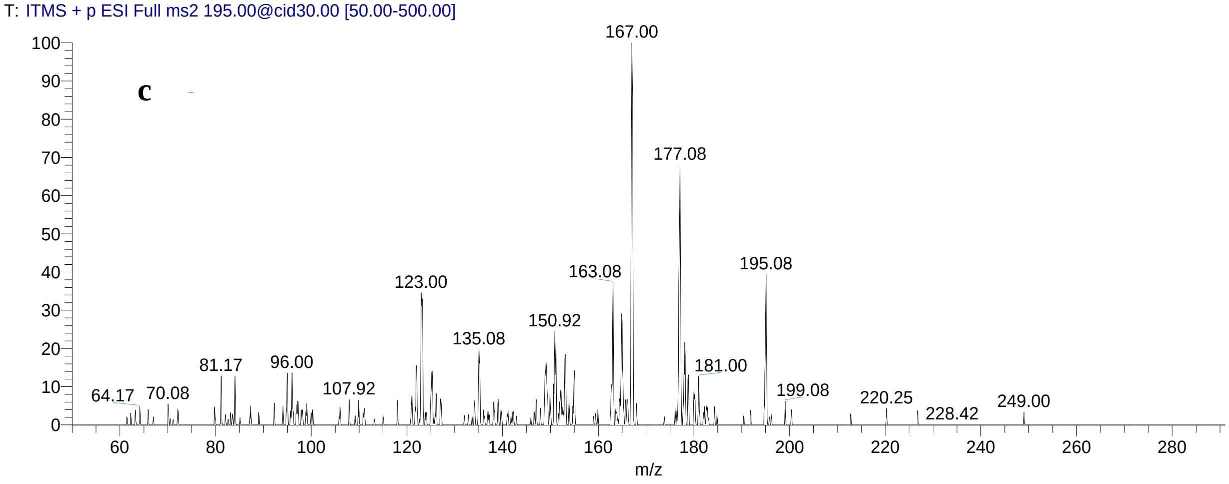

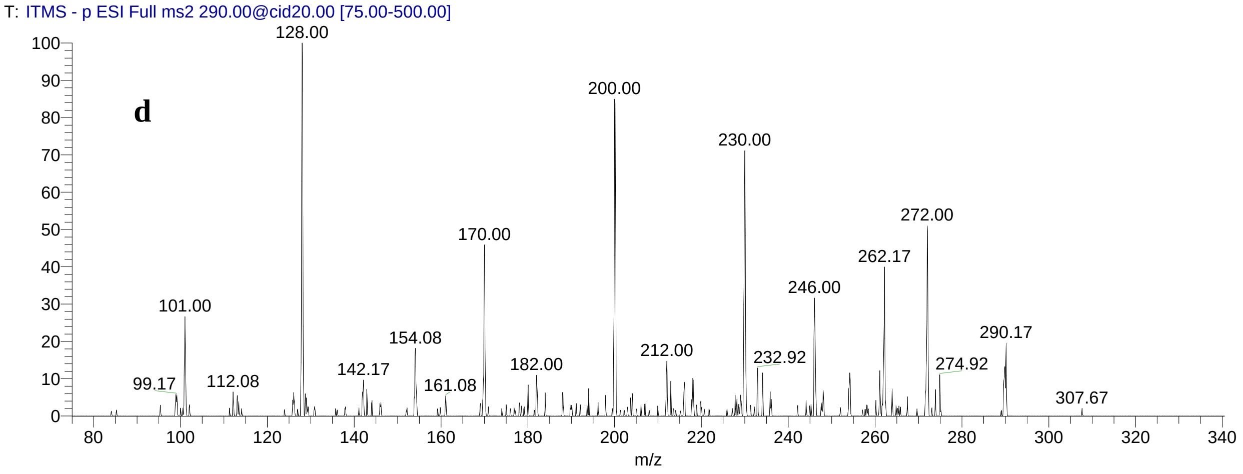
T: ITMS - p ESI Full ms2 290.00@cid20.00 [75.00-500.00]
d
128.00
200.00
230.00
272.00
262.17
246.00
170.00
101.00
154.08
290.17
212.00
232.92
142.17
182.00
274.92
99.17
112.08
161.08
307.67
m/z

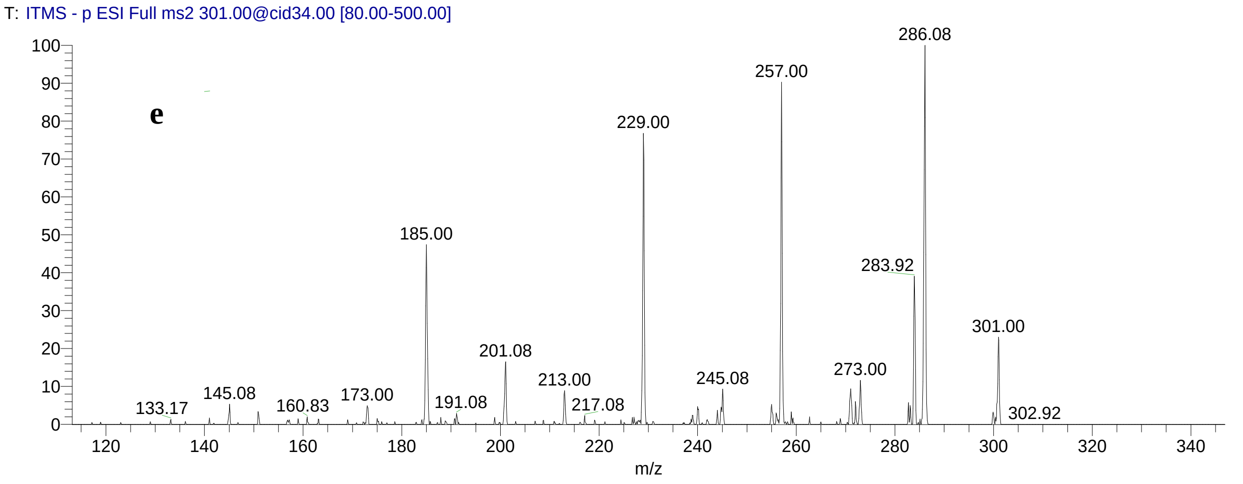
T: ITMS - p ESI Full ms2 301.00@cid34.00 [80.00-500.00]
e
286.08
257.00
229.00
185.00
283.92
301.00
201.08
245.08
273.00
213.00
217.08
191.08
173.00
145.08
160.83
133.17
302.92
m/z

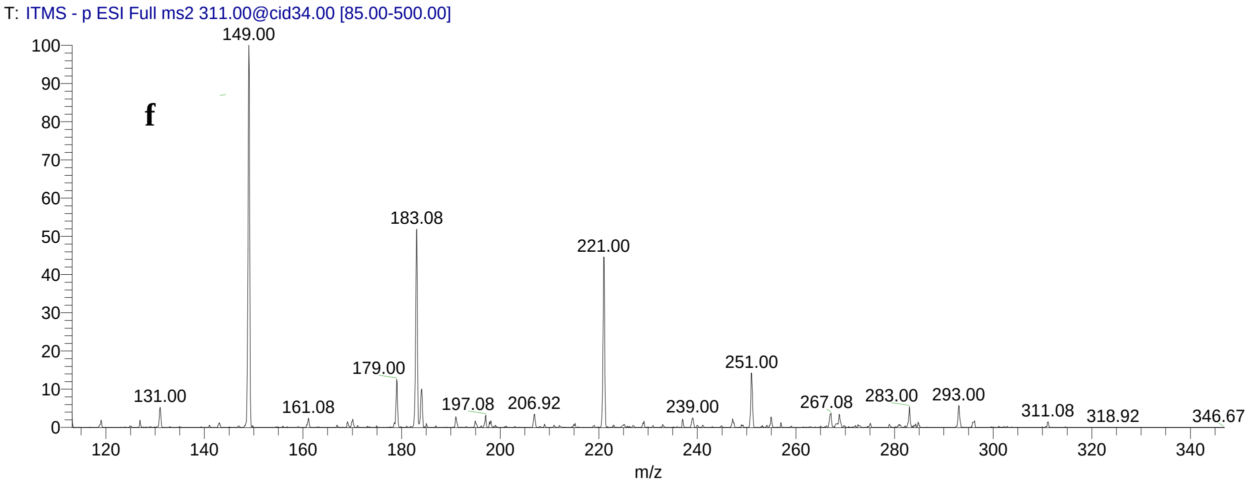
T: ITMS - p ESI Full ms2 311.00@cid34.00 [85.00-500.00]
f
149.00
183.08
221.00
251.00
179.00
131.00
161.08
197.08
206.92
239.00
267.08
283.00
293.00
311.08
318.92
346.67
m/z

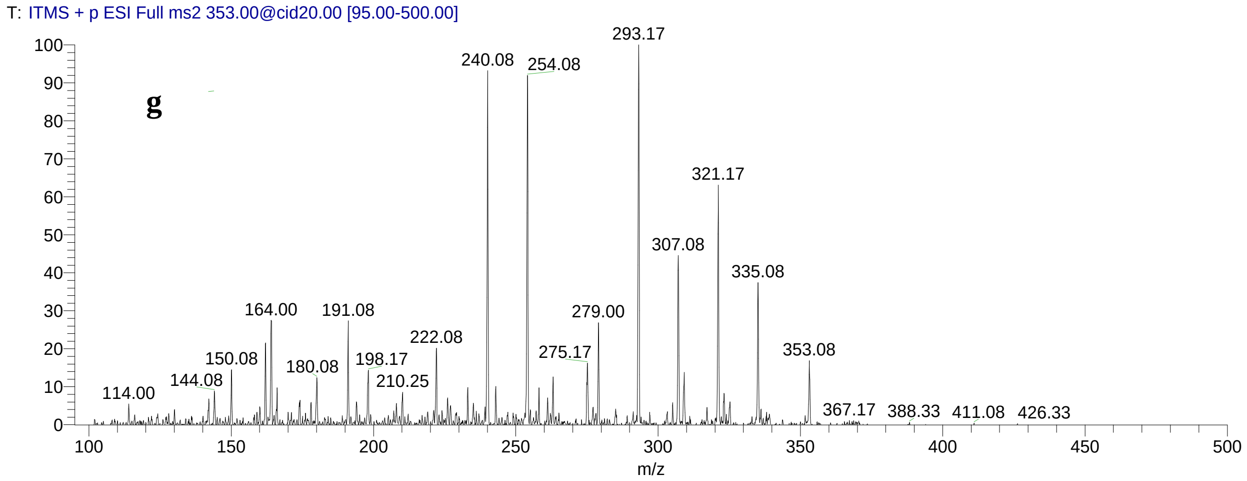
T: ITMS + p ESI Full ms2 353.00@cid20.00 [95.00-500.00]
g
293.17
240.08
254.08
321.17
307.08
335.08
164.00
191.08
279.00
222.08
353.08
150.08
198.17
275.17
180.08
144.08
210.25
114.00
367.17
388.33
411.08
426.33
m/z

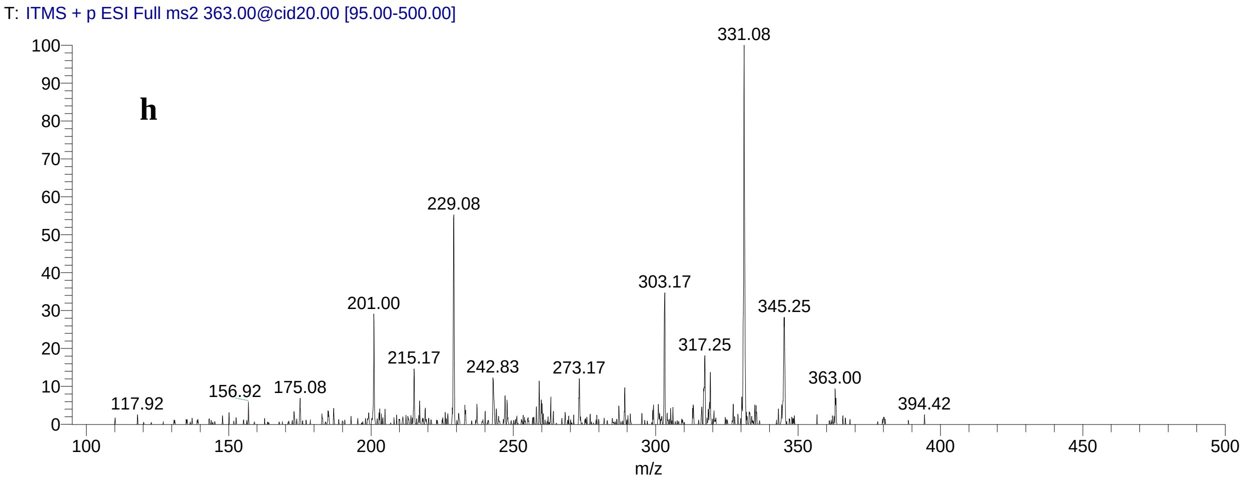
T: ITMS + p ESI Full ms2 363.00@cid20.00 [95.00-500.00]
h
331.08
229.08
303.17
201.00
345.25
317.25
215.17
242.83
273.17
363.00
175.08
156.92
117.92
394.42
m/z
