# Supplementary material for: Antiinflammatory and Anticancer Properties of Grewia asiatica Crude Extracts and Fractions: A Bioassay-Guided Approach
Source: Biomed Res Int. 2022 Mar 28;2022:2277417. doi: 10.1155/2022/2277417 (PMC8979695; doi:10.1155/2022/2277417)
Supplement: Supplementary Materials — Supplementary Figures 1 and 2: raw data of ES-MS/MS analysis of GAHAF5 and GAMF3 fractions showing spectras of known and unknown compounds. Supplementary Table 1: qualitative screening of G. asiatica fruit extracts showing presence of secondary plant metabolites. [file 2277417.f1.zip › Supplementary figure 2.pdf]

Supplementary figure 2 (a – e): ESI-MS/MS analysis of GAMF3

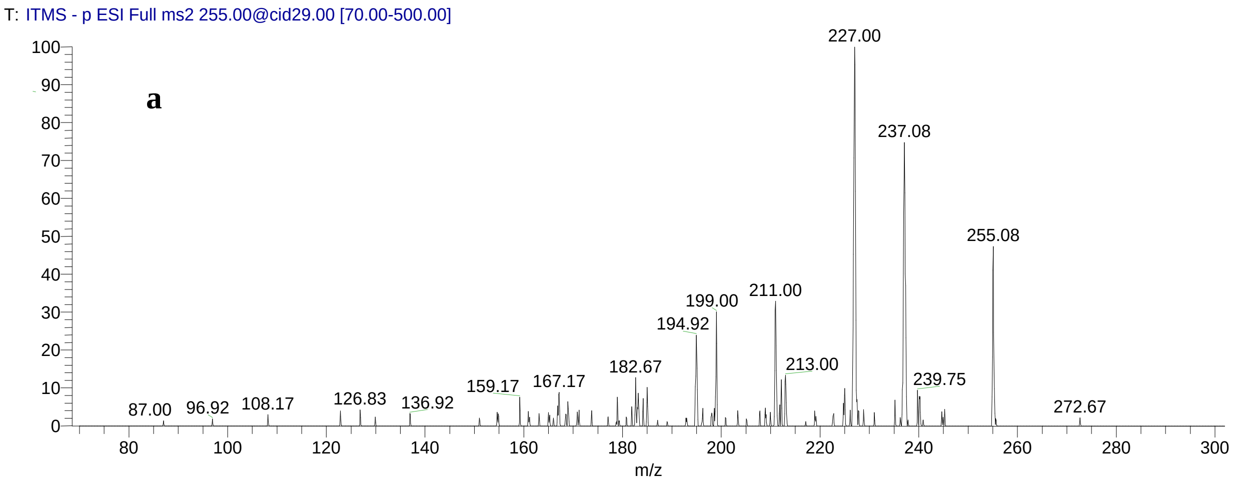

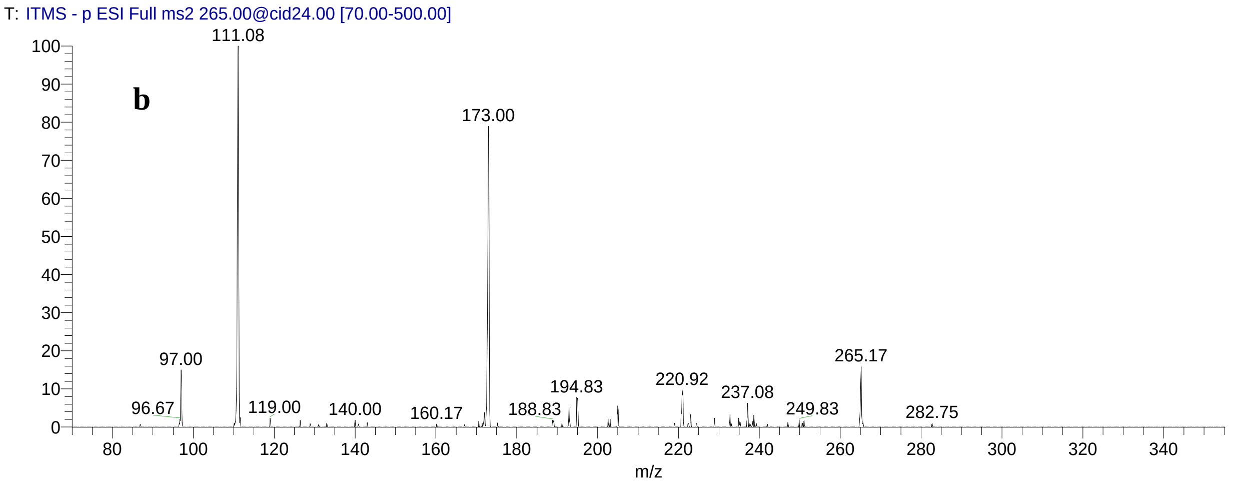

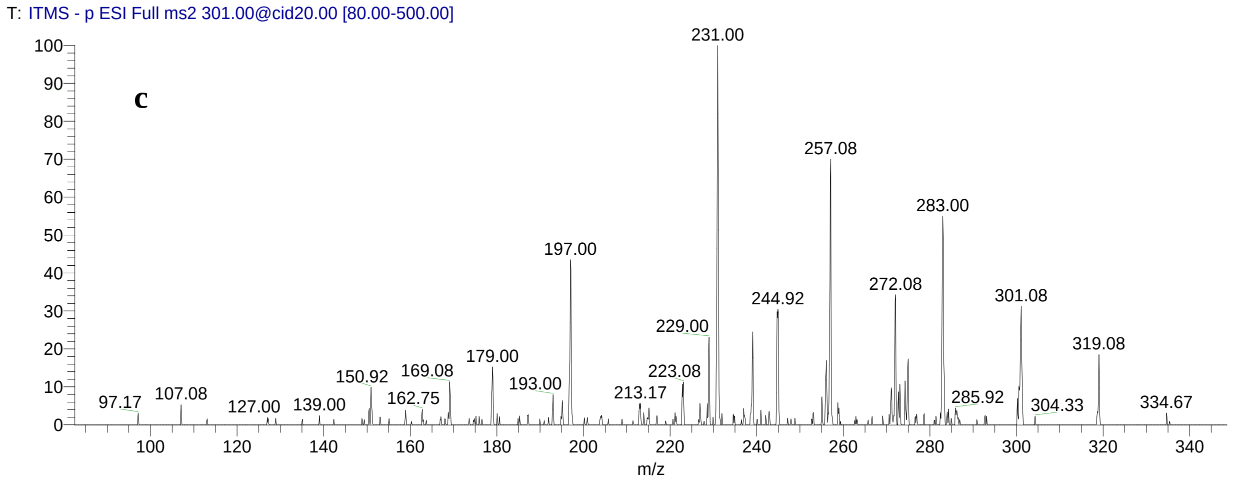

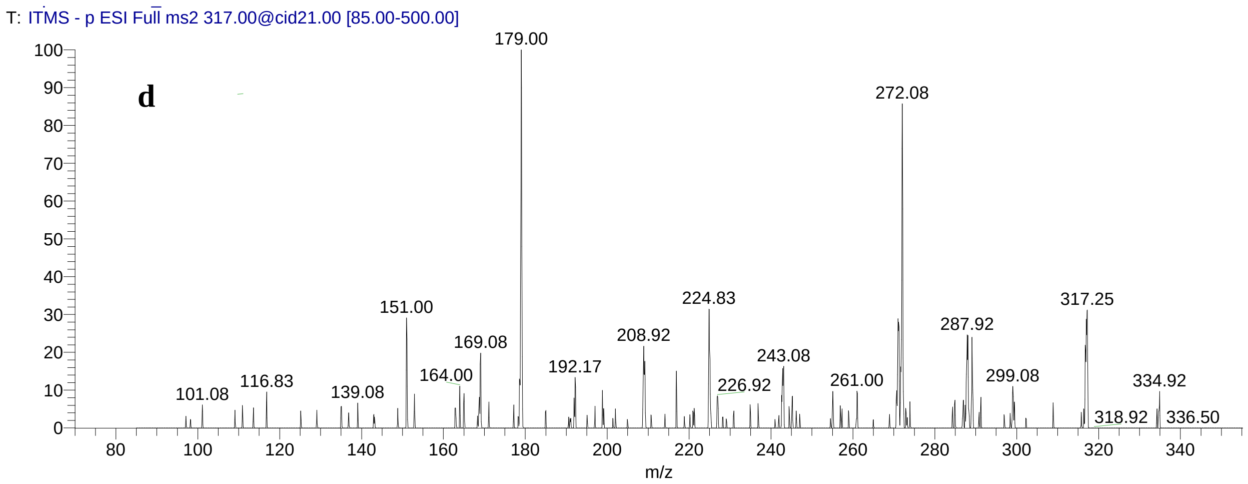

T: ITMS - p ESI Full ms2 317.00@cid21.00 [85.00-500.00]
d
179.00
272.08
151.00
224.83
317.25
287.92
169.08
208.92
243.08
192.17
164.00
226.92
261.00
299.08
334.92
116.83
101.08
139.08
318.92
336.50
m/z

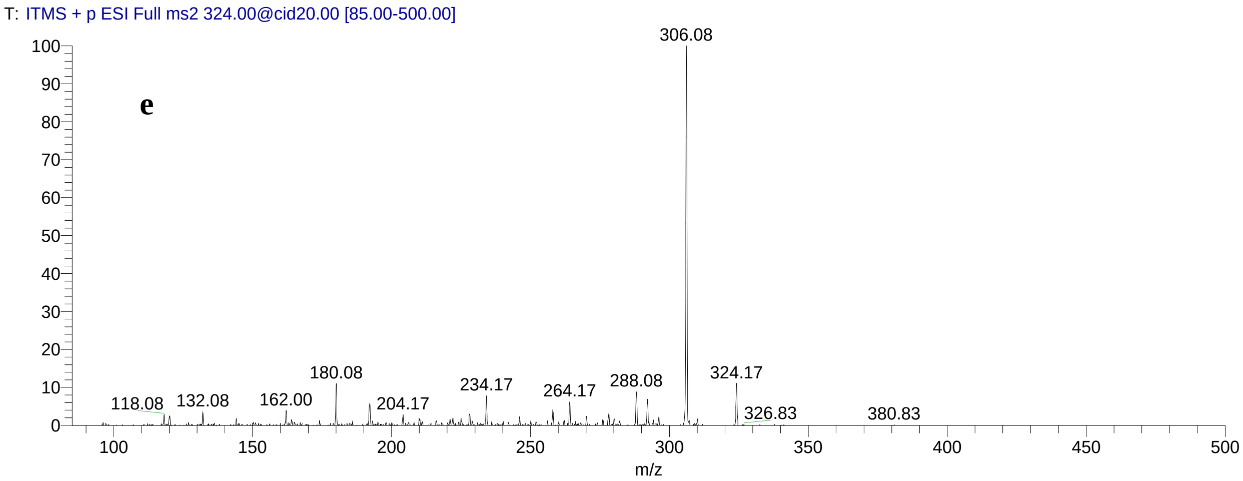

T: ITMS + p ESI Full ms2 324.00@cid20.00 [85.00-500.00]
e
306.08
180.08
324.17
234.17
264.17
288.08
118.08
132.08
162.00
204.17
326.83
380.83
m/z
