# Supplementary material for: Antiinflammatory and Anticancer Properties of Grewia asiatica Crude Extracts and Fractions: A Bioassay-Guided Approach
Source: Biomed Res Int. 2022 Mar 28;2022:2277417. doi: 10.1155/2022/2277417 (PMC8979695; doi:10.1155/2022/2277417)
Supplement: Supplementary Materials — Supplementary Figures 1 and 2: raw data of ES-MS/MS analysis of GAHAF5 and GAMF3 fractions showing spectras of known and unknown compounds. Supplementary Table 1: qualitative screening of G. asiatica fruit extracts showing presence of secondary plant metabolites. [file 2277417.f1.zip › Supplementary Table.docx]

**Supplementary Table.** Qualitative screening of secondary metabolites in G. asiatica extracts

| Secondary metabolites | Extract | Results |
| --- | --- | --- |
| Saponins | 100% MeOH | - |
|  | 50% MeOH |  |
| Flavonoids | 100% MeOH | + |
|  | 50% MeOH | + |
| Alkaloids | 100% MeOH | + |
|  | 50% MeOH | - |
| Triterpenes | 100% MeOH | - |
|  | 50% MeOH |  |
| Phenols | 100% MeOH | + |
|  | 50% MeOH |  |
| Tannins | 100% MeOH | + |
|  | 50% MeOH |  |

Absent, - ; Present, + ; MeOH – Methanol
